# The Influence of the Differentiation of Genes Encoding Peroxisome Proliferator-Activated Receptors and Their Coactivators on Nutrient and Energy Metabolism

**DOI:** 10.3390/nu14245378

**Published:** 2022-12-18

**Authors:** Agnieszka Maciejewska-Skrendo, Myosotis Massidda, Filippo Tocco, Katarzyna Leźnicka

**Affiliations:** 1 Faculty of Physical Culture, Gdansk University of Physical Education and Sport, 80-336 Gdansk, Poland; 2 Institute of Physical Culture Sciences, University of Szczecin, 71-065 Szczecin, Poland; 3 Department of Medical Sciences and Public Health, Faculty of Medicine and Surgery, Sport and Exercise Sciences Degree Courses, University of Cagliari, 72-09124 Cagliari, Italy

**Keywords:** *PPARG*, *PPARD*, *PPARG*, *PPARGC1A*, nutrient metabolism, energy metabolism, dietary intervention, exercise intervention, genetic polymorphism

## Abstract

Genetic components may play an important role in the regulation of nutrient and energy metabolism. In the presence of specific genetic variants, metabolic dysregulation may occur, especially in relation to the processes of digestion, assimilation, and the physiological utilization of nutrients supplied to the body, as well as the regulation of various metabolic pathways and the balance of metabolic changes, which may consequently affect the effectiveness of applied reduction diets and weight loss after training. There are many well-documented studies showing that the presence of certain polymorphic variants in some genes can be associated with specific changes in nutrient and energy metabolism, and consequently, with more or less desirable effects of applied caloric reduction and/or exercise intervention. This systematic review focused on the role of genes encoding peroxisome proliferator-activated receptors (PPARs) and their coactivators in nutrient and energy metabolism. The literature review prepared showed that there is a link between the presence of specific alleles described at different polymorphic points in *PPAR* genes and various human body characteristics that are crucial for the efficacy of nutritional and/or exercise interventions. Genetic analysis can be a valuable element that complements the work of a dietitian or trainer, allowing for the planning of a personalized diet or training that makes the best use of the innate metabolic characteristics of the person who is the subject of their interventions.

## 1. Introduction

A positive energy balance and weight gain result largely from a long-term mismatch between caloric intake and daily energy expenditure. The excess energy supplied is stored in the form of adipose tissue, the deposition of which in various parts of the body can lead to obesity. This is a particular threat that is becoming more and more common in highly developed societies, and is related to or directly associated with diseases of civilization such as diabetes [1], metabolic syndrome [2], primary hypertension [3], some types of cancer [4], and many others [5].

As the problems of maintaining a healthy body weight are becoming more common in the population, reduction diets are currently becoming more and more popular. The effectiveness of the diet given to a person depends on many factors. The first group is, of course, those that are directly related to the diet used: the professionalism of its planning, its composition, the ratio of nutrients supplied, the quality of the food ingredients used, etc. [6]. The second group of factors is related to the person performing the diet: their conscientiousness, lifestyle, circadian rhythm, level of physical activity, comorbidities, and many physiological variables [7] that depend on genetic factors.

The heritability of the tendency to obesity is estimated to vary widely (from 5% to 90%), depending on the research method and the hypotheses being tested [8,9]. Genetic components may play an important role, both in regulating metabolism and influencing behavioral aspects—in the presence of certain critical genetic variants, the dysregulation of energy metabolism can undoubtedly lead to an increased risk of obesity [10]. Genes code for protein products that are directly involved in the processes of digestion, assimilation, and the physiological utilization of nutrients supplied to the body [11,12]. There are also genes whose products are important factors influencing the activation and regulation of various metabolic pathways [13,14], and also determining the maintenance of the balance of metabolic changes and the shift of this balance in a specific direction [15,16]. Finally, there are the genes that code for factors that influence human behavior, eating habits, dietary preferences, etc. [17,18].

Within this group of genetic factors, many different genes have been described to date, the differentiation of which may directly or more indirectly influence the effectiveness of the reduction diets used, as well as the post-exercise weight loss [19,20,21,22,23,24,25,26]. There are many well-documented studies showing that the presence of specific polymorphic variants of these genes can be associated with more or less desirable effects of applied caloric reduction [27,28,29,30,31] and/or training intervention [32,33,34,35]. Additionally, this review will focus on these genes. We were interested in the group of genes responsible for the systemic regulation of metabolism; in particular, genes encoding peroxisome proliferator-activated receptors (PPARs) and their co-activators. A detailed review of the role of *PPAR* genes in nutrient and energy metabolism will therefore provide an overview of the impact of differentiation of genes encoding transcription factors on the efficacy of reduction diets and post-exercise weight loss.

This systematic review was prepared according to the principles of PRISMA (preferred reporting items for systematic reviews and meta-analyzes) [36]. The aim of this review is to summarize a large body of research on genes encoding peroxisome proliferator-activated receptors and their coactivators, and their role in nutrient and energy metabolism. The methods of analysis and inclusion criteria were specified in advance. This included which outcomes were of primary interest, how the reviewers extracted information about these outcomes, and the methods the reviewers used to summarize the outcome data. Study characteristics and report characteristics were used as the criteria for eligibility. The study eligibility criteria included all studies of variant genes encoding PPAR proteins, and their coactivators involved in nutrient and energy metabolism in humans. There were no restrictions on the types of participants, the types of intervention, or the types of outcome measures. Report eligibility criteria included the language of publication (English-language articles only) and publication status (published material and abstracts only). The publication date was not restricted. Studies were identified by searching electronic databases and reviewing article reference lists. This search was performed in National Center for Biotechnological Information (NCBI), i.e., in PubMed and dbSNP, GeneCards, SNPedia, Google Scholar, and Web of Science. We have used the following search terms: *PPARG*, *PPARD*, *PPARG*, *PPARGC1A*, nutrient metabolism, energy metabolism, dietary intervention, exercise intervention, genetic polymorphism, and selected rs numbers. The last search was conducted on 31 August 2022. A limited updated literature search was conducted from 18 November 2022 to 27 November 2022

From the large number of records identified in the search, approximately 20% of the records were excluded based on the eligibility criteria (the language of publication and the publication status). The retrieved records were screened based on the title and abstract, and about 35% of the records required a review of the full-text publication. To select the final studies, eligibility was independently assessed in an unblinded standardized manner by 2 reviewers. Disagreements between reviewers were resolved by consensus. As a result, 173 articles were selected for the further analyses. Data extraction forms were used by the first review author to extract data of interest from the selected studies, and the extracted data were reviewed by the second author. Disagreements were resolved via discussion between the two review authors. The extracted data were analyzed for their relevance in the context of the role of *PPAR* genes variants in human nutrient and energy metabolism, and the results of these analyses were presented in this review.

In accordance with guidelines for the nomenclature of human and mouse genes, the following principle was followed for the use of abbreviations for the names of genes and of proteins encoded by them: All gene symbols are written in italics, and the symbols of the proteins they encode are written in normal type; genes in mice and other rodents are abbreviated in italics, beginning with a capital letter, and the remainder is written in lower case (e.g., *Pparg*); proteins in mice are abbreviated in normal type, beginning with a capital letter, and the remainder are written in lower case (e.g., Pparγ); human genes are abbreviated in italics, and all abbreviations are capitalized (e.g., *PPARG*); and human proteins are abbreviated in normal script, with all letters of the abbreviation being capitalized (e.g., PPARγ) [37,38].

## 2. The PPAR Family

**Figure 1:**
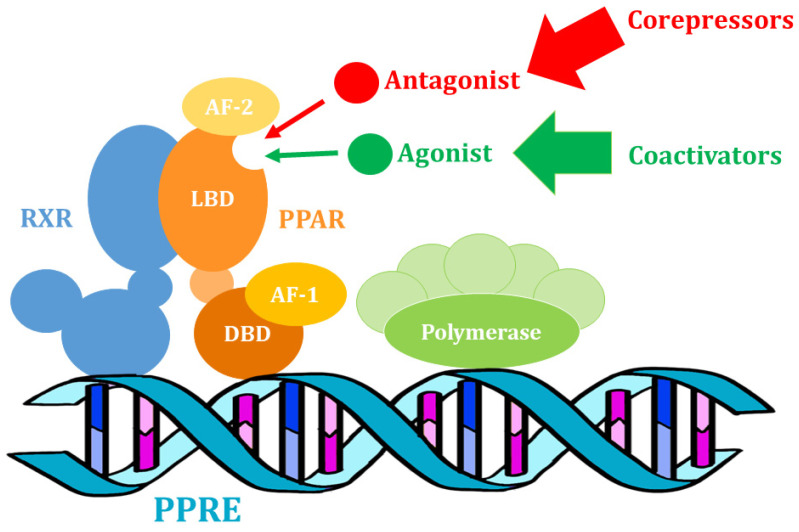
The actions of PPAR proteins: Activation of transcription of target genes involving PPAR–RXR heterodimers and coactivators (own elaboration, after: Brunmeir and Xu 2018 [46]). RXR—retinoid X receptor; PPAR—peroxisome proliferator-activated receptor; PPRE—PPAR response element; DBD—DNA binding domain; LBD—ligand binding domain; AF-1—activation function 1 domain; AF-2—activation function 2 domain.

The systemic regulation of metabolism occurs at many levels in human cells. At the deepest molecular level, metabolic flexibility depends on the configuration of many different metabolic pathways regulated by key transcription factors, many of which interact closely with each other [39]. From this point of view, transcription factors are considered as very important elements of metabolic regulatory networks. In this group, genes encoding peroxisome proliferator-activated receptors (PPAR) are one of the best studied. These proteins belong to the nuclear hormone receptor family, which in turn is part of the steroid receptor superfamily [40]. PPARs are ligand-activated receptors that, after integration, are translocated to the nucleus, where they change their structure and regulate the transcription of their target genes (Figure 1) [41]. Therefore, these receptors are transcription factors that control the activity of many genes, mostly those encoding proteins involved in lipid metabolism and regulating the balance of glucose utilization in the body [42]. Peroxisome proliferator-activated receptors can be activated both by fatty acids and their derivatives from food, and formed via de novo lipogenesis and via lipolysis [43,44]. In this way, PPARs provide balance in nutrient metabolism and maintain metabolic flexibility, which is important for lipid metabolism, glucose homeostasis, cholesterol metabolism, and other important metabolic networks [43].

The peroxisome proliferation-activated receptor family includes three subtypes: PPARα, PPARβ/δ, and PPARγ [40]. These three subtypes are encoded by distinct genes, and they differ from each other in terms of their tissue distribution, ligand specificity, and physiological role. Consistent with their expression profiles, PPARs each perform unique functions in regulating energy metabolism [45].

## 3. The *PPARG* Gene

**Figure 2:**
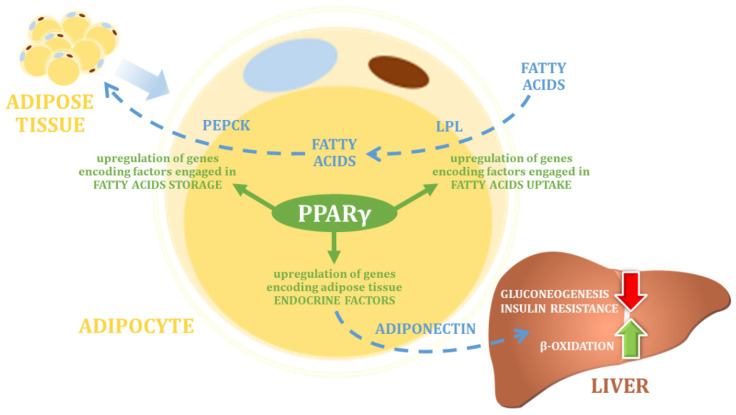
The main physiological function of PPARγ (own elaboration, after: Skat-Rørdam et al. 2018 [78]). PEPCK—Phosphoenolpyruvate carboxykinase; LPL—lipoprotein lipase.

Within the PPAR protein family, peroxisome proliferator-activated receptor gamma (PPARγ) is one of the most frequently described factors in the context of its role in regulating metabolic changes that may affect body weight changes. PPARγ protein is expressed in adipocytes (where its expression is highest) in white and brown adipose tissue, and in the large intestine and spleen. PPARγ plays a key role in the regulation of adipocyte differentiation and adipogenesis, as well as in the regulation of lipid biosynthesis and lipoprotein metabolism, and thus in the control of energy balance (Figure 2) [47]. Biochemical studies revealed that PPARγ agonists promote a flow of fatty acids into adipose tissue and away from skeletal muscle and liver under physiological conditions [48,49,50,51]. These processes lead to a decrease in fatty acid metabolism, and consequently to an increase in glucose utilization via the Randle cycle in muscles [52]. Most of these effects are mediated in a complicated network via insulin-dependent signaling pathways [53].

In humans, the PPARγ protein is encoded by the *PPARG* gene, which is located on the chromosome 3 at position 3p25.2 [54]. Analyses of the genetic expression process have shown that in the case of the *PPARG* gene, the phenomenon of the alternative reading of its sequence using different promoters (including those located within the gene) is observed, combined with the alternative splicing of primary transcripts (hnRNA), leading to the formation of different types of mature mRNA molecules (labeled with symbols from mRNAγ1 to mRNAγ7), and consequently, to the formation of many isoforms of the PPARγ protein (referred to as PPARγ1–PPARγ7) [55,56,57]. Each of these isoforms is characterized by a well-defined expression pattern, differential tissue distribution, and specific activity. The presence of so many variants of the PPARγ protein means that this transcription factor exerts a very broad range of functions in the human body.

Numerous reports suggest that the functional differentiation of the PPARγ protein may be important for maintaining a healthy body weight [58,59,60,61,62,63]. PPARγ is known to be a modulator of insulin-dependent signaling pathways—indicating that it may act through a variety of complex metabolic networks [64,65,66,67,68]. We now know with certainty that PPARγ can sensitize skeletal muscle and liver to insulin [69,70,71], but we do not know the full mechanism that is responsible for this effect. One of the most surprising observations (from a study on rodents) is that Pparγ can sensitize tissues to insulin, both via the supraphysiological stimulation of Pparγ with antidiabetic drugs, and via a decrease in Pparγ activity, resulting from a complete knockout of the *Pparg* gene [72], or (from a study on humans) via changes in its functionality due to a specific polymorphic variant of the *PPARG* gene [73]. People who are carriers of certain polymorphic forms (alleles) of the *PPARG* gene may be somewhat “metabolically burdened” because the PPARγ protein that they produce is less or more active as a stimulator of the expression of certain genes involved in fatty acid and glucose metabolism, or the regulation of insulin-dependent signaling pathways [73,74]. Indeed, *PPARG* may contribute to obesity [60] by controlling food intake and appetite, not only through changes in insulin sensitivity [72], but also by regulating the transcription of the leptin gene [75], which is a key regulator of the central control of eating behavior. Other studies suggest that the presence of certain alleles in the *PPARG* gene can lead to obesity [60], insulin resistance, and type 2 diabetes [76] or metabolic syndrome [77].

The most commonly studied polymorphic variant described as mediating nutritional effects on weight loss and maintenance is the C34G substitution (rs1801282) (Table 1), located in exon B, which is an integral part of the *PPARG* gene [79]. As a result of this nucleotide substitution at position 12 of the encoded PPARγ2 protein, alanine occurs instead of proline (Pro12Ala polymorphism). The presence of alanine at position 12 (12Ala allele) is associated with a reduced binding affinity of the PPARγ2 protein to the promoter regions of the PPARγ-controlled target genes, resulting in their much weaker activation [80,81]. The functional significance of the amino acid modification Pro12Ala, in the PPARγ2 protein, results from its localization within the PPARγ2 molecule, namely within the AF-1 domain of the amino terminus of the PPARγ2 protein, which controls ligand-independent transcriptional activity [82]. It is suggested that the Pro12Ala change in the AF-1 domain may indirectly facilitate phosphorylation and/or sumoylation processes, which reduce the potential of liganded PPARγ2 to activate the transcription of its target genes [53,83,84,85].

As mentioned above, PPARγ regulates adipocyte differentiation and controls body fat storage. Therefore, it is natural that the differentiation of the *PPARG* gene, and in particular, the functional significance of the Pro12Ala variants in relation to body weight measured by BMI and susceptibility to obesity have been the subject of numerous studies [86,87,88,89,90,91,92,93]. The widely varying results of some studies suggest that the Pro12Ala polymorphism modulates body weight [80,86,94,95], but that its effects are modified by environmental factors such as differences in dietary habits or levels of physical activity [96,97,98].

Generally, carriers of the *PPARG* 12Ala allele have significantly lower BMI values and lower fasting insulin levels with concomitant higher insulin sensitivity, as well as lower fasting plasma glucose, higher high-density lipoprotein (HDL) cholesterol, and lower total triglyceride levels [80,99,100]. It has been suggested that the lower transcriptional activity of the alanine variant of the PPARγ2 receptor, which is associated with a functional repression of the transcription of genes stimulating adipogenesis, is responsible for the lower BMI values observed in 12Ala homozygotes. The slower accumulation of adipose tissue characteristic of carriers of the 12Ala allele results in better tissue response to insulin and improved insulin sensitivity [80]. This hypothesis seems to be confirmed by the significantly increased activity of PPARγ2 receptors in obese individuals [101]. High PPARγ2 receptor activity likely shifts the balance of metabolic changes toward fat storage and BMI increase, which may be considered a significant risk factor for the potential development of obesity in carriers of the Pro12 allele. On the other hand, the decreased activity of PPARγ2 proteins reduces the risk of developing insulin resistance, and the risk of developing type 2 diabetes in carriers of the 12Ala allele [99,100]. This assumption has been confirmed in studies of people with type 2 diabetes, among whom people with the 12Ala allele are much less common [80,102].

However, differential effects of the *PPARG* 12Ala allele on BMI were found in overweight/obese and lean individuals. Normal body weight participants who were 12Ala homozygotes had lower BMIs, in both the study of Danish subjects [86] and the previous study of Finnish non-obese subjects [80]. On the other hand, 12Ala homozygotes had higher BMI values compared to Pro12 allele carriers in obese Danish participants [86], which was confirmed in the American study of Caucasians with obesity, where 12Ala allele carriers had higher BMIs compared to Pro12 homozygotes [94]. Moreover, a meta-analysis with 40 data sets from 30 independent studies with a total of 19136 subjects showed that in obese individuals, carriers of the Ala12 allele had higher BMI values than homozygotes for the Pro12 allele, whereas this effect was not observed in lean individuals [95].

It should be also noted, however, that the biological effects of Pro12Ala variants are likely to be complex and highly dependent on the interaction between diet and gene, as well as the particular ligand involved. It is worth noting that PPARγ is a ligand-activated transcription factor that is known to be activated by dietary fatty acids. Dietary fats are important modulators of *PPARG* gene transcription, leading to changes in this gene expression in response to diet. The effect of fasting was observed in mice, where it was associated with the downregulation of *Pparg* expression, whereas a high-fat diet increased *Pparg* expression in adipose tissue [103]. Consistent with these results, a low-energy diet was shown to downregulate PPARγ mRNA expression in adipocytes from obese humans [101].

There are studies that indicate an interaction between the dietary patterns of fatty acid intake and the Pro12Ala polymorphism in the *PPARG* gene. The results of one such study suggest that when diets are rich in polyunsaturated fatty acids (i.e., when the P:S ratio is high), the mean BMI is much lower in 12Ala carriers than in Pro12 homozygotes, whereas in individuals who consume large amounts of saturated fatty acids (the P:S ratio is low), 12Ala carriers have a higher mean BMI compared with Pro12 homozygotes [96]. These observations can be explained at the molecular level. Previously cited research suggests that the 12Ala variant is associated with a decreased transcriptional activity of the PPARγ2 receptor [80,81]. Polyunsaturated fatty acids are ligands for PPARγ2—if the P:S ratio is high and the supply of polyunsaturated fatty acids is large, such ligands would likely be more effective stimulators of adipogenesis in Pro12 homozygotes (characterized by more active PPARγ2 receptors) than in 12Ala carriers (with less active PPARγ2 receptors). This would be consistent with the decreasing adiposity observed in 12Ala carriers with an increasing P:S ratio [96].

In addition, the effect of dietary fatty acid intake on BMI can be influenced by physical activity. A study in nondiabetic subjects showed that the effects of diet and activity level can be modified by the Pro12Ala genotype [97]. In this study, the beneficial additive effects (described as significant reductions in fasting insulin levels) of physical activity and healthy diet (i.e., rich in polyunsaturated fatty acids) were observed mostly in *PPARG* Pro12 homozygotes. In Pro12 homozygotes, the effect achieved on lowering fasting insulin levels when only one intervention factor was included (a healthy diet without physical activity, or increased activity with an unhealthy diet) was about half as large as when both factors were included together. In 12Ala participants, the same additive relationship between diet and physical activity did not exist: fasting insulin levels were reduced only when both factors, diet and activity, were increased simultaneously. However, in contrast to the Pro12 homozygotes, no significant differences in fasting insulin levels were observed in the carriers of the 12Ala allele between completely inactive subjects with an unhealthy diet, and subjects with one of the intervention factors (a diet rich in unsaturated fatty acids or regular physical activity). The fasting insulin levels observed in all these subjects were comparable to those of Pro12 homozygotes who were completely inactive and who consumed large amounts of unhealthy saturated fat in their daily diet. However, when both the P:S dietary ratio and activity level are elevated, fasting insulin levels in 12Ala subjects are slightly lower than the lowest value observed in the Pro12/Pro12 genotype [97]. This means that in Pro12 homozygotes, at least one factor (diet or exercise) is sufficient for achieving the first visible health-promoting effects, whereas carriers of the 12Ala allele need a combination of both factors (diet and exercise) to achieve the assumed goals of the lifestyle intervention used. This assumption was confirmed in a study in which only one of the intervention factors (training) was included [98]. In this experiment, it was observed that the body mass changes observed in physically active participants were modulated by the *PPARG* Pro12Ala genotype such that carriers of the 12Ala allele were characterized by the least weight loss after training, suggesting that for carriers of this variant, training itself is not sufficient to achieve the desired effects in terms of weight loss.

All of the above data suggest that *PPARG* is a strong candidate gene that predisposes to obesity and that acts mainly by regulating adiposity, but also by influencing food intake and appetite control via modulating insulin-dependent signaling pathways. Moreover, the variation of the *PPARG* gene may play an important role in influencing the response to diet and the controls of eating behaviors. The *PPARG* Pro12Ala genotype is associated with susceptibility to obesity; however, the observed effects of its presence in an individual’s genotype are highly dependent on that individual’s lifestyle.

## 4. The *PPARD* Gene

**Figure 3:**
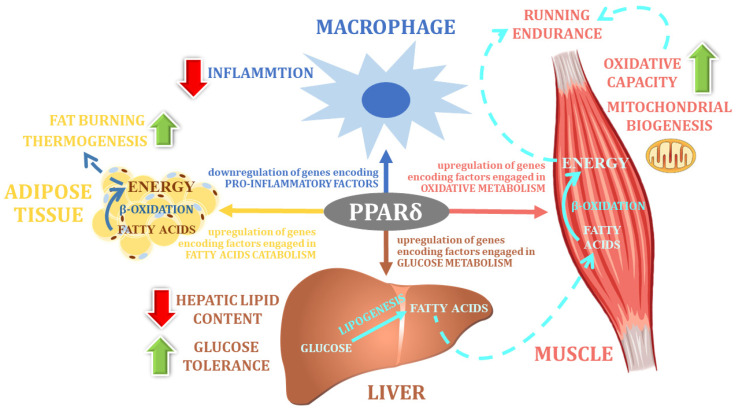
The main physiological function of PPARδ (own elaboration, after: Reilly and Lee 2008 [133]).

Post-exercise body mass changes are controlled by a large number of genes with mild to moderate individual effects. Gene networks that are associated with BMI-related sub-phenotypes revealed nearly 100 genes that are associated with BMI phenotypic variability [104]. Within the family of PPAR proteins, in addition to the previously presented PPARγ protein, another protein—peroxisome proliferator-activated receptor delta (PPARδ), encoded by the *PPARD* gene—has been described as being important in the context of its role in the effectiveness of reduction diets and post-exercise weight loss [45]. PPARδ, like other members of the PPAR family, is a transcription factor that regulates the expression of numerous genes encoding factors that are involved in the biochemical changes of most nutrients. Therefore, the PPARδ protein is considered to be one of the most important regulatory factors coordinating various metabolic pathways at the cellular level. Detailed studies show that this factor is particularly important for fatty acid catabolism and energy homeostasis (Figure 3) [105,106,107]. Indeed, in adipose tissue, the activation effect of PPARδ seems to be opposite to that of PPARγ: PPARδ stimulates fat burning, whereas PPARγ is mainly responsible for controlling fat storage [45].

The *PPARD* gene is located on human chromosome 6 at position 6p21.31 [107,108]. In contrast to *PPARA* (encoding PPARα) and *PPARG* (encoding PPARγ), the *PPARD* gene in humans is expressed in many tissues almost throughout the body [109,110]. A particularly high level of expression of the *PPARD* gene is found in tissues where intense lipid metabolism occurs: adipose tissue, heart, and skeletal muscle. In these tissues, high levels of PPARδ protein are observed, which are necessary to stimulate the expression of genes encoding factors involved in the processes of active fatty acid uptake by cells and the β-oxidation of fatty acids [111].

The activation of PPARδ factor correlates with a decrease in the plasma triglyceride levels, resulting in their lower availability and slower storage in adipose tissue [112]. This suggests that proper PPARδ protein activity may be associated with a reduced susceptibility to obesity. This suggestion seems to be confirmed by studies with transgenic animals. The overexpression of the mouse *Ppard* gene in adipose tissue resulted in lean mice that did not develop obesity, even when fed a high-fat diet [113]. Detailed analyses in these animals showed that the Pparδ protein increased the expression of genes encoding factors involved in catabolic changes in fatty acids and adaptive thermogenesis. On the other hand, mice with a completely blocked *Ppard* gene showed a marked tendency toward obesity [113]. A further study with synthetic ligands activating the Pparδ protein in mice treated with a high-fat diet showed that the effect of such specific Pparδ agonists leads to the inhibition of body weight gain, but without effects on the appetite of the experimental animals and the amount of food consumed [114]. This suggests the possibility of using such synthetic PPARδ agonists as potential therapeutic agents for the treatment and/or prevention of obesity—however, due to the highly pleiotropic effect of PPARδ activation [105,115], their potential therapeutic use in humans still requires much research.

A number of single nucleotide polymorphisms (SNPs) have been described in the human *PPARD* gene (Table 1), and they have been analyzed with respect to their potential significance for various human traits [116]. Such phenotypic modulation, due to the presence of specific polymorphic variants of the *PPARD* gene, has been described in numerous scientific publications [117,118,119,120,121,122,123,124,125,126,127].

The most commonly studied SNP in the context of the efficacy of dietary interventions or weight changes after exercise is rs2016520, which is located outside the region of the *PPARD* gene encoding the PPARδ protein, in the regulatory region of exon 4, approximately 87 base pairs upstream of the translation start site. An in vitro study indicated that the presence of the C allele at the rs2016520 site is associated with a higher transcriptional activity of the *PPARD* gene compared to the T allele, as confirmed through electrophoretic mobility shift assays [128]. Given the role of the PPARδ protein in lipid metabolism, it can be assumed that the increased transcriptional activity of the *PPARD* gene leading to higher levels of PPARδ protein will have an impact on the levels of lipid fractions observed in blood serum in carriers of different sequence variants of the *PPARD* gene. Indeed, there are reports indicating that carriers of the rs2016520 C or T allele exhibit subtle differences in plasma LDL and HDL cholesterol, and in apoB and triglyceride concentrations [128,129,130,131]. It has also been shown that the presence of additional polymorphic variants described at other sites in the *PPARD* gene (rs6902123 in intron 2, rs2076167 in exon 7, and rs1053049 in exon 9) correlates with insulin-dependent glucose uptake by cells, and this effect appears to be exclusive to skeletal muscle, as no such correlation was observed for adipose tissue [132].

Intervention studies have also been conducted, analyzing the effects of the presence of different variants of the *PPARD* gene on the changes in various biochemical parameters observed in response to the training intervention. One of these studies showed that, with respect to the previously mentioned point rs2016520, a significant reduction in total cholesterol was observed in carriers of the C allele after training, with a concomitant reduction in blood triglycerides [134]. The influence of two other polymorphic points in the *PPARD* gene was also analyzed: rs2267668 in intron 3 and rs1053049 in exon 9. Carriers of the rs2267668 G allele showed a significant decrease in total cholesterol, whereas AA homozygotes at the same point showed a significant decrease in triglyceride levels. An opposite trend was observed in TT homozygotes at point rs1053049, which were reported in the groups with the highest triglyceride levels after training. The analyzes of the haplotype systems reconstructed for the above three SNPs in the *PPARD* gene show that the G/C/T haplotype (rs2267668/rs2016520/rs1053049) is the least favorable system in terms of achieving the desired effects after training, as the lowest body weight losses after training intervention were observed in carriers of this haplotype [134].

In another large-scale exercise and dietary lifestyle intervention program (Tuebingen Lifestyle Intervention Program), the effects of polymorphisms in intron 2 (rs6902123), intron 3 (rs2267668), and exon 9 (rs1053049) of the *PPARD* gene on body composition changes in subjects participating in such a dual intervention were studied. Detailed analyses indicate that carriers of the rarer alleles described in all polymorphic points studied are characterized by a smaller increase in relative muscle volume, and a smaller decrease in adipose tissue mass and fat storage in the liver, suggesting that these rarer allele forms may be responsible for the development of insulin resistance and an increased risk of developing type 2 diabetes [135]. Another study of participants in the same intervention program also found that carriers of the rs2267668 G allele responded most poorly to the applied training, as determined by individual anaerobic threshold (IAT) and peak aerobic capacity on a cycloergometer [136]. A subsequent intervention study conducted at the same time and including a dietary and exercise intervention, demonstrated that the association between a high-fat diet and the risk of developing metabolic syndrome could be modulated by the *PPARD* rs2016520 genotypes: individuals with moderate fat consumption who were carriers of the C allele had the lowest risk of developing metabolic syndrome [137].

Regarding the association between *PPARD* gene differentiation and obesity, numerous polymorphic SNPs have been studied, but the results presented in the literature on this topic are inconclusive. On the one hand, there are reports from Korea of a positive correlation between the presence of the major alleles of rs2016520 and rs1053049 in the *PPARD* gene and of higher body mass index in their carriers [138]. Moreover, analyses of the adjacent polymorphic point in the *PPARD* gene showed that the G allele at SNP rs2076167 was overrepresented in the obese subgroup [139]. Study conducted in the Polish population showed that carriers of the C allele at rs1053049 and the G allele at rs2267668 had an increased risk of obesity [123]. On the other hand, there has also been a study conducted in other large population of middle-aged white subjects where no association between SNPs in the *PPARD* gene and obesity-related phenotypes was found [140].

In summary, in the case of several polymorphic variants that have been described and tested in the *PPARD* gene, their modulatory effects on the efficacy of dietary and exercise interventions appears to be reasonably well documented. However, it is extremely difficult to specify a clear positive system of allelic variants in the *PPARD* gene that would provide its carrier with the greatest efficacy of a reduction diet, or the chance of the greatest weight loss through increased physical activity. At this point, it must be emphasized that the functional significance of most of the polymorphic points mentioned above (with the exception of rs2016520) is currently unknown. Therefore, to better understand the interrelationships between the individual components of the haplotype systems constructed for the *PPARD* gene, more detailed molecular studies are required to explain the functional significance of each of the polymorphic variants in the *PPARD* gene described so far.

## 5. The *PPARA* Gene

**Figure 4:**
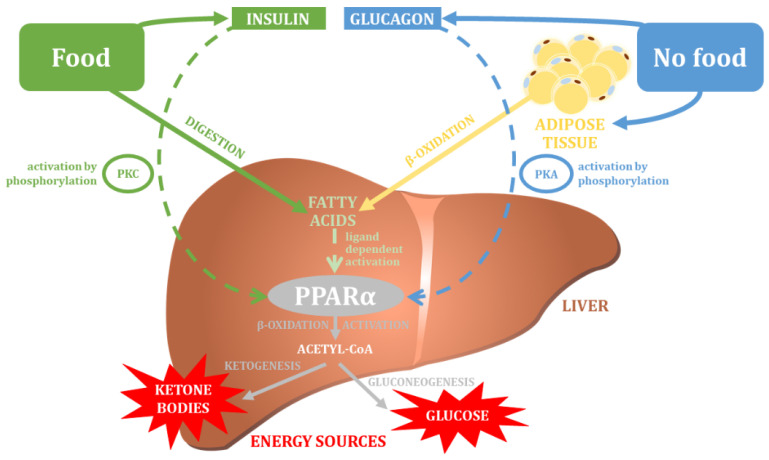
The main physiological function of PPARα (own elaboration, after: Pawlak et al. 2015 [150]). PKC—protein kinase C; PKA—protein kinase A.

Peroxisome proliferator-activated receptor alpha (PPARα) is the last member of the PPAR family of proteins that undoubtedly plays a crucial role in the systemic regulation of metabolism. The PPARα protein is mainly expressed in liver, but also in brown adipose tissue, kidney, and cardiac and skeletal muscle [141]. The main physiological function of PPARα is the regulation of fatty acid transport and their β-oxidation, which is an important element in maintaining the balance of lipid metabolism in the human body (Figure 4) [43,142]. More specifically, the active PPARα protein inhibits lipid accumulation by decreasing the rate of glycolysis and increasing glycogen synthesis and the aerobic metabolism of fatty acids [143].

Detailed analysis shows that PPARα protein stimulates the expression of the *APOA5* gene, encoding apolipoprotein A5 (Apo-AV), which lowers blood triglyceride levels [144]. The activation of PPARα decreases the plasma levels of triglyceride-rich lipoproteins primarily by stimulating expression of the *LPL* gene, which encodes a lipoprotein lipase that hydrolyzes lipoprotein triglycerides [145]. PPARα also causes an increase in high-density lipoprotein cholesterol (HDL-C), with a concomitant decrease in low-density lipoprotein cholesterol (LDL-C) [146]. The PPARα factor mediates the increase in HDL-C levels by stimulating the expression of genes encoding apolipoproteins Apo-AI and Apo-AII [147]. Furthermore, the activation of PPARα in intestinal cells decreases cholesterol esterification, inhibits chylomicron production, and increases HDL synthesis by enterocytes [148].

The effect of PPARα varies depending on the nutritional state of the organism. During fasting, the PPARα protein stimulates the expression of genes encoding fatty acid transport proteins, thereby facilitating the uptake by the liver cells of free fatty acids released from adipose tissue as a result of lipolysis processes taking place there [149,150]. PPARα activity has also been shown to promote fatty acid oxidation in the mitochondria, and peroxisomes in liver cells [43,149]. During long periods of fasting, PPARα stimulates the formation of ketone bodies, which are used as an emergency power source in tissues outside the liver. The PPARα protein shows a slightly different effect under conditions of adequate nutrition: it is then a factor that promotes de novo lipogenesis, which involves the transfer of precursors for fatty acid synthesis (acetyl-CoA) from mitochondria to the cytosol, and aims to obtain the energy (in the form of liver triglycerides) needed to survive periods of starvation [149]. Thus, the nutritional state of the organism determines the activity of the PPARα factor, which in a well-nourished organism has an activity that is related to the coordination of lipogenesis processes, while in conditions of starvation it acts as a factor that promotes the uptake of fatty acids and their oxygen conversion. It is worth mentioning that nutritional status also affects PPARα protein activity by controlling PPARα-phosphorylating kinases: during full nutrient intake, PPARα activity is stimulated by insulin-activated MAPK (mitogen-activated protein kinases) and glucose-activated PKC (protein kinase C), whereas during fasting periods, PPARα is activated by glucagon-activated PKA (protein kinase A) and AMPK (5′AMP-activated protein kinase) [150].

Considering the importance of PPARα in the context of the effect of this transcription factor on the efficacy of applied reduction diets and the effectiveness of weight reduction after exercise, it should be noted that the activation of PPARα stimulates weight reduction in rodents [151], but that this effect has not been clearly confirmed in humans—keeping in mind that studies that do not confirm this phenomenon have so far been conducted mainly in patients with type 2 diabetes [152,153], and that there are no such studies in healthy humans. On the other hand, there is also a study suggesting that the activation of PPARα in the central nervous system can lead to increased food intake [154], but there is also evidence of the opposite effects, where the activation of PPARα-dependent pathways has been shown to induce satiety and reduce appetite [155].

The *PPARA* gene is located on human chromosome 22 at position 22q13.31 [156]. Many SNP points have been identified within the human *PPARA* gene (Table 1) [157]; one of the most frequently described being the C-to-G transversion, the consequence of which is an amino acid substitution at position 162 (Leu162Val; rs1800206) of human PPARα [158,159]. This amino acid change has real functional significance because it occurs in the region of the *PPARA* gene that encodes the DNA-binding domain, namely, the second zinc finger. The conversion of valine to leucine occurs directly adjacent to the cysteine that is required to coordinate the Zn^2+^ atom of the second zinc finger, i.e., upstream of the region that determines the specificity and polarity of PPARα binding to DNA in the target genes [160]. There are evidences indicating that the variant of the PPARα protein with valine at position 162 (allele 162Val) is a more active transcription factor than the form with Leu162 [161]. However, it should be emphasized that this functional diversity depends on the concentration of PPARα ligands, which may be, for example, free fatty acids. In the absence of or at low concentrations of ligands, the activity of the 162Val variant is almost half that of the leucine form. In contrast, at high ligand concentrations, the activity of the valine form increases significantly and may even exceed that of the Leu162 variant [161].

There is ample evidence that the Leu162Val polymorphism plays a role in shaping lipid metabolism. Several studies suggest that the presence of the 162Val allele is positively correlated with elevated total cholesterol, LDL-C, triglyceride, and Apo-B levels, which translates into an increased risk of hyperlipidemia and obesity [158,162,163,164]. On the other hand, there are reports that do not confirm the association between Leu162Val polymorphism and body mass index, body fat composition, liver fat content, or more generally with obesity [165,166].

As with *PPARG* gene diversity, the functional significance of the *PPARA* polymorphic variants may be altered by various environmental factors, including diet. An intervention study suggested that carriers of the 162Val allele have higher levels of triglycerides and Apo-CIII, but only if their diet was low in polyunsaturated fatty acids. The opposite effect was observed when the diet was enriched in polyunsaturated fatty acids [167]. Similar effects were found in another study, which showed that carriers of the 162Val allele had a lower total cholesterol and Apo-AI levels in diets rich in polyunsaturated fatty acids [168].

Another polymorphic point in the *PPARA* gene that is of functional significance is Val227Ala (rs1800234), which appears to be present primarily in individuals of oriental descent [169,170]. In this case, the Val/Ala amino acid substitution, which is a consequence of the nucleotide change in the *PPARA* gene, occurs in the so-called hinge region, which is located between the DNA-binding domain (DBD) and the ligand-binding domain (LBD). The dimerization domain of the protein is located in this region, and therefore, the conversion of valine to alanine at position 227 is thought to be of functional importance: the 227Ala variant is characterized by a lower transactivation activity than the Val227 form in the presence of PPARα-specific ligands [171]. An in vitro study suggested that the 227Ala allele is associated with a slower transcription of the cytochrome P-450 4A6 gene and the mitochondrial 3HMG-CoA synthase gene [171]. In addition, the alanine variant is positively correlated with reduced levels of cholesterol, LDL-C, and triglycerides [169,170]—thus, the 227Ala allele is considered as being important for lipid lowering and protection against the development of steatosis [172]. The Val227 variant, on the other hand, is associated with higher cholesterol levels—an effect that occurs primarily in individuals who do not drink alcohol. In alcoholic drinkers, the 227Ala allele appears to be associated with increased gamma-glutamyl transpeptidase activity [173].

Among noncoding SNPs, the most commonly studied is the point in intron 7 of the *PPARA* gene, which is a G-to-C transversion (rs4253778) [174,175]. Although this is a non-functional polymorphism (causing no changes in the amino acid sequence of the encoded protein), there are reports indicating that the presence of specific alleles at this SNP point is associated with changes in the expression of the *PPARA* gene itself, such that the C allele correlates with a lower expression of the *PPARA* gene in its carriers [176], which in turn may lead to a weaker stimulation of the expression of genes controlled by PPARα [177]. CC homozygotes have significantly higher total and LDL cholesterol levels [178], and the C allele showed a positive association with dyslipidemia [179]. On the other hand, carriers of the C allele were found to have significantly lower TG and VLDL levels and higher HDL-C levels in populations of different ethnic origins [180].

Another noncoding SNP worth mentioning is the T in A nucleotide change in the intron of the *PPARA* gene, designated rs4253747. This change appears to be of particular interest from the point of view of the efficacy of the reduction diets used, as it has been shown that carriers of the A allele are characterized by a much stronger appetite suppression response that occurs under certain conditions, described as high-altitude appetite loss, which is a common symptom of acute mountain sickness [181]. Exposure to high altitude is thought to stimulate *PPARA* gene expression [182,183]—the involvement of rs4253747 variants in this process is not yet known, but it is expected that they may be important for binding transcription factors within the *PPARA* gene promoter or enhancers, such that they control the expression of the *PPARA* gene itself [181].

There are also polymorphic points in the *PPARA* 3′UTR region (the gene region encoding the so-called untranslated region), such as rs6008259 and rs3892755, for which clear interactions between the presence of specific variants, diet, and metabolic parameters have been demonstrated. With respect to the SNP rs6008259, it has been shown that higher levels of total cholesterol and LDL-C are observed in carriers of the G allele at a high daily intake of linolenic acid, compared with carriers of the genotype AA. Similarly, carriers of the C allele described in item rs3892755 with a high daily intake of eicosapentaenoic acid and docosahexaenoic acid are characterized by high levels of total cholesterol and LDL-C, compared with carriers of the TT genotype [157,184].

The PPARα protein is undoubtedly an important metabolic regulator. As mentioned earlier, the diversity of the *PPARA* gene affects the content of different lipid fractions and related particles observed in the blood plasma of carriers of different polymorphic variants. The sequence variants described at the different polymorphic sites of the *PPARA* gene can therefore be considered as promising markers that could potentially be used to predict the effects of appropriate nutritional interventions.

## 6. The *PPARGC1A* Gene

Coordinated metabolic regulation is undoubtedly one of the key factors in maintaining a healthy body weight. PPARs are an important element of this regulation, but they are not the only important factors influencing BMI and related values. Specific proteins, known as coactivators, are required for the activity of PPARs and many other transcription factors. One such coactivator is peroxisome proliferator-activated receptor gamma coactivator-1-alpha (PGC-1α), which in humans is encoded by the *PPARGC1A* gene [185].

**Figure 5:**
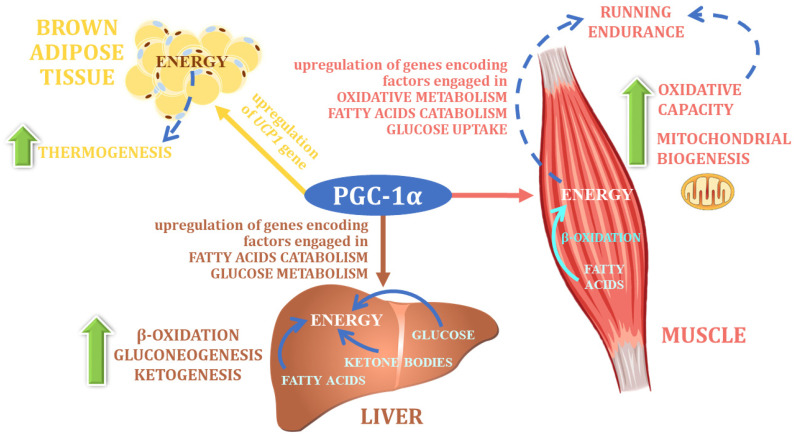
The main physiological function of PGC-1α (own elaboration, after: Cheng et a. 2018 [16]). UCP1—uncoupling protein 1.

PGC-1α is considered to be the central regulator of energy metabolism (Figure 5). It was originally described as a cold-inducible coactivator of nuclear receptors associated with adaptive thermogenesis, as the first study on this factor indicated that Pgc-1α mRNA expression is dramatically increased upon cold exposure of mice, in both brown fat and skeletal muscle, tissues that represent the main centers of thermogenesis in mammals [186]. PGC-1α has been shown to control oxidative metabolism in many cell types [187]. The expression of the *Ppargc1a* gene encoding this factor in mice is induced under the influence of physical activity in muscle, where it stimulates mitochondrial biogenesis [188], angiogenesis [189], and the transformation of muscle fibers toward their greater oxygen capacity [190]. Changes in the metabolic profile observed in the muscles of physically active individuals due to increased expression of the *PPARGC1A* gene include not only increased capillarization, muscle fiber remodeling, or mitochondrial biogenesis, but also the pleiotropic control of many pathways of energy substrate conversion, such as fatty acid oxidation, tricarboxylic acid cycle (TCA), and glucose transport pathways, as well as a significant increase in the level and rate of oxidative phosphorylation [187].

Contrary to its name, PGC-1α is not only a PPARγ coactivator, but also other transcription factors that are involved in the control of the expression of numerous key genes, not only in terms of mitochondrial biogenesis or angiogenesis, but also in terms of adipose tissue metabolism and the potential risk of developing obesity. In the late 1990s, Puigserver et al. found that obesity is the result of an imbalance between energy production (derived from calories consumed with food) and energy expenditure [186]. Total energy expenditure includes: basal metabolic rate, dietary energy expenditure, the energy cost of physical activity, and what is known as adaptive (non-shivering) thermogenesis [191]. The latter process refers to energy that is expended in response to changing environmental conditions, particularly when the body is exposed to cold or excessive caloric intake (diet-induced thermogenesis). Adaptive thermogenesis is an important component of energy homeostasis, and it is considered as being part of the metabolic defense against obesity [186].

Long-standing research has shown that PGC-1α is the master regulator of uncoupling protein 1 (UCP1) expression, which activates thermogenic programs in brown adipose tissue and elsewhere [192]. PGC-1α not only directly controls the expression of UCP-1 protein, but also stimulates the expression of several products secreted by muscle during exercise. One of these products is type I membrane protein, a precursor of irisin, which strongly stimulates the expression of UCP-1 and the functional conversion of white adipose tissue cells into brown fat cells [193]. These processes lead to a significant increase in total energy expenditure, mainly due to an increase in the component related to adaptive thermogenesis, which translates into a lower tendency towards increased weight gain, and a lower risk of obesity. This assumption seems to be confirmed by a study with transgenic mice, in which the muscular expression of the Pgc-1α protein was increased—a prolonged lifespan and resistance to age-related obesity and diabetes were observed [194]. Therefore, it can be assumed that carriers of the allelic variants of the *PPARGC1A* gene that favor a higher expression of this gene and an increased level of PGC-1α protein are characterized by a higher energy expenditure that is associated with thermogenic changes, and consequently, by a lower probability of weight gain and the development of obesity. It is worth mentioning that the thermogenic function of PGC-1α is similar to the effect of PPAR-δ mentioned above.

The *PPARGC1A* gene was fully described in the late 1990s [185,186]. Since then, numerous polymorphic variants of this gene have been described in humans, including those of functional significance. The most commonly described polymorphic point in the *PPARGC1A* gene is the G-to-A transition at position +1564 in exon 8 (rs8192678), which is known as the Gly482Ser substitution (Table 1) [195]. Studies with different groups of participants suggest that the 482Ser allele is associated with a reduction in *PPARGC1A* gene expression, resulting in lower mRNA levels in carriers of this variant [196,197,198]. In vitro experiments suggest that the amino acid change at position 482 at the molecular level decreases the ability of PGC-1α to bind to the MEF2C protein, which is a transcription factor that is involved in controlling the expression of many genes [199]. It has been shown that the efficiency of the interaction between coactivator and transcription factor is significantly reduced when serine is present at position 482 (in the domain responsible for the interaction between PGC-1α and MEF2C) instead of glycine [199,200]. In mice, the activation of Mef2c by Pgc-1α protein has been shown to play an autoregulatory role, as the Pgc-1α/Mef2c complex enhances expression of the gene encoding mouse Pgc-1α protein [201].

In the context of metabolic importance, the major human gene controlled by MEF2C is the *GLUT4* gene, which encodes glucose transporter 4. The GLUT4 protein is an insulin-sensitive glucose transporter in muscle and adipose tissue that stimulates glucose uptake into cells in response to the binding of insulin to a corresponding receptor on the cell membrane surface [202,203]. The size of the GLUT4 protein pool in muscle or adipose tissue is thought to be one of the factors determining the insulin sensitivity of a given tissue. This assumption is confirmed by a study in transgenic mice, in which the expression of the human *GLUT4* gene was artificially induced—increased insulin sensitivity and increased glucose uptake by muscle cells were observed in these animals [204]. A cell culture study suggested that the effective stimulation of *GLUT4* gene expression involving the MEF2C factor requires its interaction with the PGC-1α coactivator [200]. In the case of the PGC-1α-serine protein variant, a lower efficacy of the MEF2C interaction on the *GLUT4* gene is observed, as well as a weaker insulin-dependent response and a decrease in glucose consumed by cells [199,200], which in turn impairs glycogen synthesis and consequently lipid metabolism, and it has the potential to disrupt glucose–lipid homeostasis [195,205,206].

Numerous studies have indicated that carriers of the 482Ser allele have been shown to have a significantly higher risk of developing type 2 diabetes [119,195,207,208], which may be a consequence of the decrease in insulin secretion observed in carriers of this 482Ser variant [196,197]. In addition, the presence of the 482Ser form was associated with some impairment of aerobic capacity, which was particularly acute in studies of professional athletes [209,210,211,212,213] and of physically active individuals who are not professional athletes, where the best response to aerobic exercise was observed in carriers of the Gly482 allele [136].

The 482Ser variant has also been associated with an increased incidence of dyslipidemia [195,205,206], and an increased risk of obesity in different populations [205,214,215]. Initially, the importance of *PPARGC1A* gene diversity in the context of obesity development was confirmed in genome-wide association studies. The first of these, a genome-wide study of abdominal fat via computed tomography in the Québec Family Study, revealed that in the human genome, the diversity of the chromosomal region where the *PPARGC1A* gene was mapped was associated with subcutaneous abdominal fat and the propensity for fat storage in the abdominal depot [216]. The same chromosomal region (4p15.1) has been linked with BMI in Mexican Americans [217].

In subsequent years, research focused on examining the functional significance of polymorphic variations such as Gly482Ser in relation to an increase in BMI and the risk of obesity. In the Austrian study of middle-aged men and women, the association of the *PPARGC1* gene Gly482Ser locus with obesity was found only in women, suggesting that this locus may contribute to genetic susceptibility to multifactorial obesity disease [205]. The next study was devoted to evaluating the relationships between the Gly482Ser variant of *PPARGC1A* with non-esterified fatty acid content, and to describing the effect-modifying role that adipose mass might have on the relationships between Gly482Ser and fatty acid levels in healthy Europids. The authors of this study reported that non-esterified fatty acid levels were higher in carriers of the minor 482Ser allele during glucose loading and that the effects of the allele on fatty acid levels were additive [215].

The impact of the Gly482Ser polymorphism in *PPARGC1A* on body composition and glucose tolerance, as well as insulin sensitivity and secretion, was demonstrated in a Finnish study of nondiabetic offspring of patients with type 2 diabetes. It was found that the 482Ser variant (in a specific haplotype block with other variants studied) was associated with increased glucose levels in an oral glucose tolerance test [218]. This was confirmed in the Doetichem cohort study, in which the presence of the 482Ser variant was associated with higher glucose levels in overweight or obese individuals [219]. The same trend was observed in another study of severely obese individuals: significantly higher fasting plasma glucose levels in obese participants carrying the Ser482 allele [220]. On the other hand, a much stronger statistical association was observed in the study by Povel et al., with significantly lower glucose levels in normal-weight individuals. Given that a strong association between glucose and *PPARGC1A* genotypes was present only in lean subjects, it could be suggested that the action of the *PPARGC1A* gene, which is involved in both fatty acid oxidation and glucose metabolism, is modified by BMI [219].

At this point, it is worth noting that the aforementioned associations observed between elevated non-esterified fatty acid levels in carriers of the 482Ser allele were most pronounced in obese individuals [215]. In addition, *PPARGC1A* expression is significantly decreased in the white adipose tissue of obese individuals compared to lean individuals [221]. It has been suggested that the downregulation of *PPARGC1A* in obesity may be related to elevated non-esterified fatty acid levels [215], which was confirmed in the intervention study by Hoeks et al. [222].

It is likely that this effect is particularly enhanced in obese carriers of the 482Ser allele due to the weaker activation of *PPARGC1A* gene expression associated with this variant allele. This assumption was confirmed in a study that found a significantly higher frequency of 482Ser homozygotes and 482Ser alleles in the group of obese participants—therefore, it was concluded that the 482Ser variant may be a risk factor for the development of obesity in the Korean female population [214]. An association between the presence of the 482Ser allele and increased body weight has also been observed in younger individuals. Albuquerque et al. observed an association between the *PPARGC1A* allele 482Ser, and body weight and waist circumference in Portuguese children [223].

Given the physiological role of the PGC1-α protein, which plays a critical role in maintaining glucose, lipid, and energy homeostasis, and acts as a master regulator of energy expenditure [16,187,224,225], the Gly482Ser polymorphism in the *PPARGC1A* gene may influence energy balance and may be a plausible candidate for investigating its importance in the efficacy of reduction diets and post-exercise weight loss.

## 7. Conclusions

PPAR proteins, as transcription factors, are involved in regulating the genetic expression of hundreds of genes associated with various metabolic pathways. This makes genes encoding PPAR proteins ideal candidates for studying the effects of genetic diversity on complex metabolic regulation. Overall, a large body of research demonstrates that there is a link between the presence of specific alleles described at different polymorphic points in *PPAR* genes, and various human body characteristics that are crucial for the efficacy of nutritional and/or exercise interventions.

As shown in the prepared review of the literature, inconclusive results were found in some cases, but it is possible to indicate some variants of *PPAR* genes for which their importance in relation to the effectiveness of reduction diets has been well documented. An analysis of the presence of such variants can be a valuable element that complements the work of a dietitian or trainer, and that facilitates the planning of a personalized diet or training that allows for the best use of the innate metabolic characteristics of the person who is the subject of their interventions. The use of genetic testing is now routine in the work of professionals dealing with athletes, for example, but it is also becoming increasingly popular with people who are simply interested in learning about the molecular determinants that determine how their bodies respond to caloric restriction and physical activity.

This is part of the currently very dynamically developing trend of nutrigenomic research, the aim of which is to personalize nutrition for the individual, paying special attention to the goals of a particular person (weight reduction, improving the quality of life, increasing physical performance, preventing certain diseases, etc.). The approach used includes dietary recommendations indicating which ingredients should be restricted, eliminated, or their proportion in the daily diet increased, depending on the presence of certain polymorphic variants in the genome of the person tested.

The major limitation of this method is the still limited knowledge about the role of individual genes in shaping body weight, depending on the regulation of metabolism and other characteristics of the human body (eating habits, receptor sensitivity, etc.). The studies cited in this and other systematic reviews were conducted on relatively small groups of participants, sometimes from different ethnic backgrounds, and using different research methods. Therefore, the group of genes for which we have sufficiently convincing evidence in this area is not very large, yet genes from the PPAR family seem to be strong candidates in this group.

At this point, it should be mentioned that the group of genes used in the context of dietary recommendations should include not only genes that are responsible for the systemic regulation of metabolism (such as genes from the PPAR family), but also a number of other genes, e.g., genes that are responsible for nutrition and appetite, and for controlling the achievement of satiety levels, genes encoding receptors that are related to the perceptions of tastes and odors, or genes related to food intolerances. A failure to include such a broad spectrum of genes in nutrigenomic dietary recommendations can lead not only to unsatisfactory effects of the applied diet, but in extreme cases, even to the occurrence of health complications as a result of an inappropriate diet.

In summary, until recently, the use of genetic testing to provide highly personalized dietary recommendations for specific individuals seemed to be something of a scientific fantasy. Today, thanks to a significant reduction in the cost of molecular testing procedures, advances in research on the effects of genetic variants on individual physiological responses, and a significant increase in the public awareness of the role of genetic factors in regulating the functioning of the human body—the use of genetic testing by not only physicians, but also nutritionists and sports coaches, seems to be an inevitable evolutionary path of their daily practice in working with patients, clients, and athletes.

## Figures and Tables

**Table 1:** Selected polymorphic variants of genes encoding PPAR family proteins, and their molecular significance and phenotypic effects (details in the text).

Protein	Gene	RsID	Alleles (Variants)	Molecular Significance	Selected Phenotypic Effects
PPARγ	*PPARG*	rs1801282	C (Pro12)	Normal binding affinity of the PPARγ2 protein to the target genes	Lower BMI values in obese individuals or when diet is rich in saturated fatty acids
			G (12Ala)	Reduced binding affinity of the PPARγ2 protein to the target genes	Lower BMI values in lean individuals or when diet is rich in polyunsaturated fatty acids
PPARδ	*PPARD*	rs2016520	T	Normal transcriptional activity of the *PPARD* gene	
			C	Higher transcriptional activity of the *PPARD* gene	Lower total cholesterol and blood triglycerides after training, lower risk of metabolic syndrome with moderate fat consumption
		rs2267668	A	Not known	Lower triglyceride levels after training
			G	Not known	Lower total cholesterol after training, weaker response to physical training as measured by individual anaerobic threshold and peak aerobic capacity
		rs1053049	T	Not known	Higher triglyceride levels after training
			C	Not known	Lower triglyceride levels after training
PPARα	*PPARA*	rs1800206	C (Leu162)	Higher transcriptional activity of the *PPARA* gene in the absence or at low concentrations of ligands	Lower levels of triglycerides and Apo-CIII when diet is low in polyunsaturated fatty acids
			G (162Val)	Higher transcriptional activity of the *PPARA* gene at high ligand concentrations	Lower total cholesterol and Apo-AI levels when diet is rich in polyunsaturated fatty acids
		rs1800234	T (Val227)	Normal transactivation activity of the PPARα protein in the presence of PPARα-specific ligands	higher cholesterol levels in individuals who do not drink alcohol
			C (227Ala)	Lower transactivation activity of the PPARα protein in the presence of PPARα-specific ligands	Reduced levels of cholesterol, LDL-C, and triglycerides, increased gamma-glutamyl transpeptidase activity in alcoholic drinkers
		rs4253778	G	Normal expression of the *PPARA* gene	
			C	Lower expression of the *PPARA* gene, weaker stimulation of expression of genes controlled by PPARα	Higher total and LDL cholesterol levels; positive association with dyslipidemia, lower TG and VLDL levels, and higher HDL-C levels in populations of different ethnic origins
		rs4253747	T	Not known	Weaker appetite suppression response in high-altitude appetite loss
			A	Not known	Stronger appetite suppression response in high-altitude appetite loss
		rs6008259	G	Not known	Higher levels of total cholesterol and LDL-C at a high daily intake of linolenic acid
			A	Not known	Lower levels of total cholesterol and LDL-C at a high daily intake of linolenic acid
		rs3892755	C	Not known	Higher levels of total cholesterol and LDL-C at a high daily intake of eicosapentaenoic acid and docosahexaenoic acid
			T	Not known	Lower levels of total cholesterol and LDL-C at a high daily intake of eicosapentaenoic acid and docosahexaenoic acid
PGC-1α	*PPARGC1A*	rs8192678	G (Gly482)	Normal transcriptional activity of the *PPARGC1A* gene, normal binding affinity of the PGC-1α protein to the target transcriptional factors	Normal insulin secretion; normal insulin-dependent response; lower risk of dyslipidemia, obesity and type 2 diabetes; normal glucose uptake by cells; improvement of aerobic capacity
			A (482Ser)	Lower transcriptional activity of the *PPARGC1A* gene, reduced binding affinity of the PGC-1α protein to the target transcriptional factors	Decreased insulin secretion; weaker insulin-dependent response; higher risk of dyslipidemia, obesity and type 2 diabetes; decrease in glucose uptake by cells; impairment of aerobic capacity

## Data Availability

Not applicable.
